# Modulation of Cell Signaling Pathways in Silica Nanoparticle-Saturated Macrophages

**DOI:** 10.3390/pharmaceutics18030344

**Published:** 2026-03-11

**Authors:** Sushanto Kumar Saha, Cansu Umran Tunc, Nitish Khurana, Philip J. Moos, Hamidreza Ghandehari

**Affiliations:** 1 Utah Center for Nanomedicine, University of Utah, Salt Lake City, UT 84112, USA; sushantokumar.saha@utah.edu (S.K.S.); cansu.tunc@utah.edu (C.U.T.); nitish.khurana@utah.edu (N.K.); philip.moos@pharm.utah.edu (P.J.M.); 2 Department of Biomedical Engineering, University of Utah, Salt Lake City, UT 84112, USA; 3 Department of Molecular Pharmaceutics, University of Utah, Salt Lake City, UT 84112, USA; 4 Department of Pharmacology and Toxicology, University of Utah, Salt Lake City, UT 84112, USA

**Keywords:** silica nanoparticles, nanotoxicology, macrophage saturation, nanomedicine, cell signaling pathways

## Abstract

**Background/Objectives**: Upon systemic delivery, macrophages take up a significant portion of nanoparticles and may become saturated. The saturation of macrophages may pose risks to overall immune function and signaling pathways. While some information is available on the survival and functionality of macrophages upon saturation with nanoparticles, there is limited understanding of the molecular-level changes that can occur and their corresponding influences on macrophage phenotypes, gene expression, and immune signaling pathways. **Methods**: In this study, RAW 264.7 macrophages were saturated with silica nanoparticles (SNPs) of different sizes (50 and 100 nm), porosities (nonporous, mesoporous), densities (solid, mesoporous, and hollow), and surface compositions (hydrophobicity) at their maximum non-toxic concentrations. The saturated macrophages were evaluated for changes in gene expression and immune signaling pathways by RNA sequencing, weighted gene co-expression network analysis (WGCNA), and Hallmark and KEGG pathway analyses. **Results**: Our results show that in the range studied, the particle size did not have a significant effect on the gene expression profile. Porous SNPs of comparable sizes resulted in increased and unique changes in the gene expression profile compared to nonporous SNPs. Major immune signaling pathways, including TNF-alpha signaling via NF-κB pathways, mTORC1 signaling, and p53 pathways, were modulated in SNP-saturated macrophages. This modulation depended on the physicochemical properties of the particles. The Th1/Th2 multiplex immunoassay revealed that the uptake of SNPs increases the amount of the TNF-alpha cytokine compared to the nontreated controls, whereas no changes in IL-6 and IL-12p70 pro-inflammatory cytokines were observed. **Conclusions**: Our results demonstrate that physicochemical properties of SNPs, such as porosity, size, surface functionality, and density, influence the modulation of gene expression and macrophage immune signaling pathways. These results, along with others, can provide guidance on the selection of silica nanoparticles for the safe and effective systemic delivery of bioactive agents.

## 1. Introduction

Silica nanoparticles (SNPs) have shown potential for the delivery of bioactive and imaging agents [1,2,3]. Being “Generally Recognized as Safe” (GRAS) by the United States Food and Drug Administration, SNPs have found usage as excipients in food and cosmetic applications [4]. The ease of synthesis, tunable physicochemical properties, stability, and ability to load cargo provide opportunities for the use of SNPs in therapeutic delivery applications [1,2,3,5,6]. SNPs have shown an improved oral bioavailability of drugs [5]. They have also been used for the intravenous delivery of imaging agents. However, to date, there are no FDA-approved products incorporating SNPs as carriers for systemic drug delivery. Concerns include limited knowledge about SNP accumulation in the reticuloendothelial organ-resident macrophages, the impacts on their immune functionality, and the potential for short- and long-term toxicity [1,7,8].

As an integral part of the innate immune system, circulating and tissue-resident macrophages take up a significant portion of systemically administered nanoparticles, potentially saturating macrophages. The saturation of macrophages by SNPs or other nanoparticles may influence their phenotype, gene expression, and immune signaling. Besides having primary functions in immunomodulation, phagocytosis, and antigen presentation [9], macrophages play a crucial role in maintaining iron homeostasis, tissue repair, and other metabolic processes [10,11]. Therefore, a detailed investigation is warranted regarding the downstream effects upon the accumulation of SNPs in saturated macrophages.

Previous studies have reported SNP-related pro-inflammatory responses, cytotoxicity, and genotoxicity [1,7,8,12,13,14,15]. These responses were shown to depend on the concentration of particles used and their physicochemical properties, including size, shape, geometry, surface charge, surface functionalization, porosity, as well as dosing frequency and incubation time [1,7,8,12,13,14,15,16,17]. In a recent study, we saturated RAW 264.7 macrophages and demonstrated that at maximum nontoxic concentrations, there were no major changes in the survival and functional activity of the RAW 264.7 macrophages [18]. While these results are promising, a more detailed understanding of the subtle gene expression profiles of macrophages in response to SNP uptake, as a function of physicochemical properties, is warranted.

To gain a better understanding of the molecular-level changes and potential sub-toxic responses, it is essential to investigate the alterations in gene expression, cell signaling behavior, and molecular pathway changes in macrophages upon SNP saturation. Previously, we studied alterations in the global gene expression, as well as time- and dose-dependent changes in RAW 264.7 macrophages and showed that the porosity of the nanoparticles plays the predominant factor in influencing gene expression [19,20]. In the present study, we expanded this investigation to include nonporous 50 and 100 nm SNPs (SNP50 and SNP100), mesoporous 100 nm SNPs (MSNPs), and hollow mesoporous 100 nm SNPs (HMSNPs) and assessed the gene expression and key pathway modulations at maximum nontoxic concentrations, aiming to study the genetic-level changes at SNP-saturated macrophages.

## 2. Materials and Methods

### 2.1. Synthesis and Characterization of SNPs

Nonporous Stöber 50 nm and 100 nm SNPs (SNP50 and SNP100) were synthesized following a modified Stöber method. Mesoporous SNPs (MSNPs) were synthesized using a surfactant-based template method. Hollow mesoporous SNPs (HMSNPs) included a three-step synthesis and a solid Stöber core synthesis, followed by a mesoporous coating. The SNPs were etched to obtain a hollow structure. The details of the synthesis methods and materials for the four different SNPs have been reported previously [18]. The SNPs were characterized for their size and morphology using transmission electron microscopy (TEM) (JEOL-JEM 1400, Peabody, MA, USA) and nanoparticle tracking analysis (NTA) (NTA 3.4 Build 3.4.4, Malvern, UK). The structure, morphology, and pore size of the SNPs were evaluated using scanning–transmission electron microscopy (STEM) (JEOL-JEM 2800, Peabody, MA, USA). The Limulus Amebocyte Lysate (LAL) assay (ThermoFisher Scientific, Waltham, MA, USA) was used to quantify the endotoxin concentration in the nanoparticle solution [18].

### 2.2. Cell Culture

The RAW 264.7 macrophage cell line was used throughout the study and was obtained from ATCC (Manassas, VA, USA). The cells were cultured in RPMI media supplemented with 10% FBS. The cell lines were authenticated using a Mouse Short Tandem Repeat Profiling Kit from ATCC (Manassas, VA, USA).

### 2.3. Cellular Uptake

A 24-h cell viability study was performed via the CCK-8 assay with a range of SNP concentrations (6.25 µg/mL to 800 µg/mL) to determine the maximum nontoxic concentrations of the SNPs on the macrophages, and the saturation timepoint for maximum internalization was determined by ICP-MS, as described in the previously published article [18].

To visualize the internalization of SNPs inside the cells, 5 × 10^5^ cells per well were grown overnight in a six-well plate and treated with the maximum nontoxic concentrations of SNPs (50 µg/mL, 25 µg/mL, 200 µg/mL, and 200 µg/mL for SNP100, SNP50, MSNP, and HMSNP, respectively) for 6 h as the saturation timepoint. The cells were fixed using a 2.5% formaldehyde/glutaraldehyde fixative solution at room temperature and then collected and centrifuged to obtain cell pellets, which were stored at 4 °C overnight. The cell pellets were resin-embedded, and ultrathin sections were obtained using a microtome knife. Transmission electron microscopy images of cells were taken using JEOL JEM-1400 microscope (Peabody, MA, USA).

### 2.4. RNA Extraction

RAW 264.7 macrophages were seeded on a six-well plate (5 × 10^5^ cells/well) and grown overnight. The cells were dosed at SNP maximum nontoxic concentrations (50 μg/mL for SNP100, 25 μg/mL for SNP50, 200 μg/mL for both MSNPs and HMSNPs, and no SNP treatment for control) and incubated for 6 h (saturation time). The total RNA of the cells was collected in RNase-free water using the Qiagen RNeasy Mini Kit (Qiagen, Hilden, Germany). The sample was treated with DNase to remove DNA and stored at −80 °C. The RNA concentration was measured with a Qubit RNA HS Assay Kit (Fisher Scientific #Q32855, ThermoFisher Scientific, Waltham, MA, USA). RNA quality was evaluated with an Agilent Technologies RNA ScreenTape Assay (5067-5579 and 5067-5580) (Agilent Technologies, Santa Clara, CA, USA). All samples were ensured to have an RNA quantity of over 25 ng/µL and an RNA integrity number (RIN) between 9 and 10 before sequencing.

### 2.5. mRNA Sequencing and Data Acquisition

RNA sequencing was performed at the High Throughput Genomics Core at the University of Utah. The library was generated using the NEB Next Ultra II Directional RNA Library Prep with Poly(A) mRNA Isolation kit (New England Biolabs, Ipswich, MA, USA) for Illumina sequencing. The sequencing was performed using the Novaseq X Reagent kit (Illumina, San Diego, CA, USA) for 150 × 150 base pair sequencing, with more than 30 million read pairs for each sample. The *Mus musculus* genome (GRCm39) was used for mapping the reads.

### 2.6. Bioinformatic and Statistical Analysis

Differential gene expression analysis was performed using the DESeq2 package (v1.42.1) in R (v4.4.2) [21], following standard workflows for count-based RNA-seq data. The genes with fewer than 10 counts were removed, and regularized log transformation (rlog) was applied to stabilize variance across samples for visualization and downstream analysis. The shrinkage of log2 fold changes with the apeglm method was used to identify differentially expressed genes [22].

To assess functional enrichment, we used the Enrichr package (v3.4) [23]. Enrichment analysis was conducted against multiple curated databases, including MSigDB Hallmark 2020, KEGG 2019 Mouse, and Gene Ontology categories for Biological Process (2025 versions). Enrichment results were visualized and interpreted to identify biological pathways and processes associated with differentially expressed genes.

Co-expression network analysis was subsequently performed using the Weighted Gene Co-expression Network Analysis (WGCNA) package (v1.73) [24]. A soft-thresholding power of 18 was used for scale-free topological assessment, as well as a “signed” structure for network analysis. The resulting gene-centric network modules were used to explore gene co-regulation and subjected to Enrichr-based pathway analysis.

### 2.7. Th1/Th2 Cytokine Analysis

In total, 2.5 × 10^5^ cells/well were seeded in a six-well plate and allowed to grow for 24 h. The cells were dosed with nontoxic SNP concentrations. After 6 h, the cell culture supernatants were collected and centrifuged at 10,000 rpm for 5 min to eliminate the residual cells. The supernatant was used to analyze Th1/Th2 cytokine panels (IFN gamma, IL-4, IL-5, IL-6, IL-12p70, TNF alpha) using ProcartaPlex mouse essential Th1/Th2 panel 6-plex (ThermoFisher Scientific, Waltham, MA, USA). Unidentified patient plasma samples were used as positive controls to validate the findings from the ELISA.

The experiments were performed with each group replicated at least three times (*n* = 3) and presented as the mean ± standard deviation (SD). The variance between the two groups was analyzed using the Student’s t-test in Microsoft Excel and GraphPad Prism Software (Version 10.1,2), with a threshold of significance set at *p* < 0.05. The graphs were drawn using GraphPad Prism Software (Version 10.1,2).

## 3. Results

### 3.1. Synthesis and Characterization

**Figure 1:**
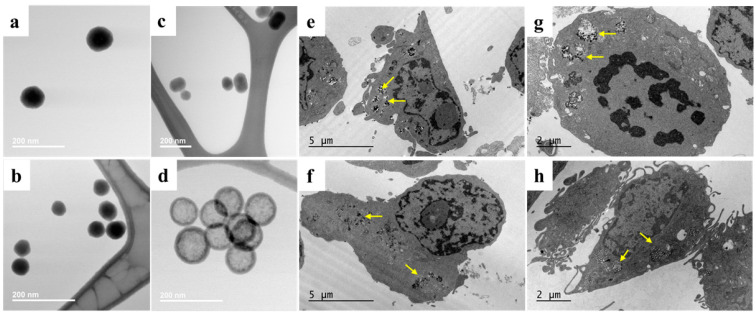
Transmission Electron Microscopy images of (**a**) SNP100, (**b**) SNP50, (**c**) MSNPs, and (**d**) HMSNPs. Images show the internalization of the SNPs in RAW 264.7 cells: (**e**) SNP100, (**f**) SNP50, (**g**) MSNPs, (**h**) HMSNPs via TEM imaging. The cells were treated with SNPs at respective maximum nontoxic doses, and no visual cellular damage was observed. The yellow arrows indicate the particles inside the cells.

The size and morphology of the synthesized SNPs were characterized using STEM and are shown in Figure 1a–d. SNP50 and SNP100 represent nonporous, spherical solid nanoparticles of 50 and 100 nm diameters on average, respectively. MSNPs are mesoporous SNPs, whereas HMSNPs are hollow mesoporous SNPs of approximately 100 nm average diameters. The diameters of the SNPs calculated with ImageJ (v1.54f) (National Institute of Health, MD, USA) for 200 nanoparticles were 55.65 ± 5.98, 126.89 ± 16.93, 115.56 ± 31.50, and 101.16 ± 10.23 nm, for SNP50, SNP100, MSNP, and HMSNPs, respectively [18]. The diameters measured by nanoparticle tracking analysis were 67.2 ± 22.3, 93.1 ± 25.7, 120.4 ± 32.5, and 97.0 ± 30.8 for SNP50, SNP100, MSNP, and HMSNPs, respectively. The pore size of the MSNP is 1.71 ± 0.22 nm and the HMSNP is 1.81 ± 0.32 nm, as determined by transmission electron microscopy [18]. The SNP solutions were checked for the absence of endotoxin contamination and showed less than 0.1 EU/mL, corresponding to 0.1 EU per mg of SNPs. The TEM images in Figure 1e–h show the cellular uptake of the SNPs. The morphology of the macrophages showed no visual damage to the cells at the respective doses. SNPs were taken up and internalized in vacuole-like structures, and no SNPs were seen in the nuclei of the cells.

### 3.2. Gene Expression Analyses

**Figure 2:**
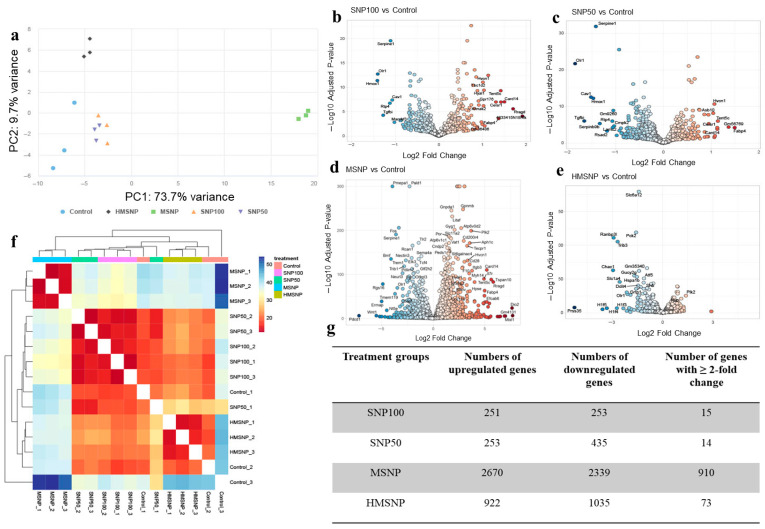
Gene expression changes in the SNP-saturated macrophages. (**a**) Principal Component Analysis (PCA) plot showing the variances between different treatment groups. Volcano plots showing the changes in the gene expression profile upon SNP saturation: (**b**) SNP100 vs. control, (**c**) SNP50 vs. control, (**d**) MSNP vs. control, (**e**) HMSNP vs. control. High-resolution images are presented in Supplementary document S1 (Figures S5–S8). (**f**) Gene expression heatmap showing the overall gene expression differences. (**g**) The table shows the number of upregulated and downregulated genes (padj < 0.05), with the number of genes with more than a twofold change compared to the nontreated control.

**Figure 3:**
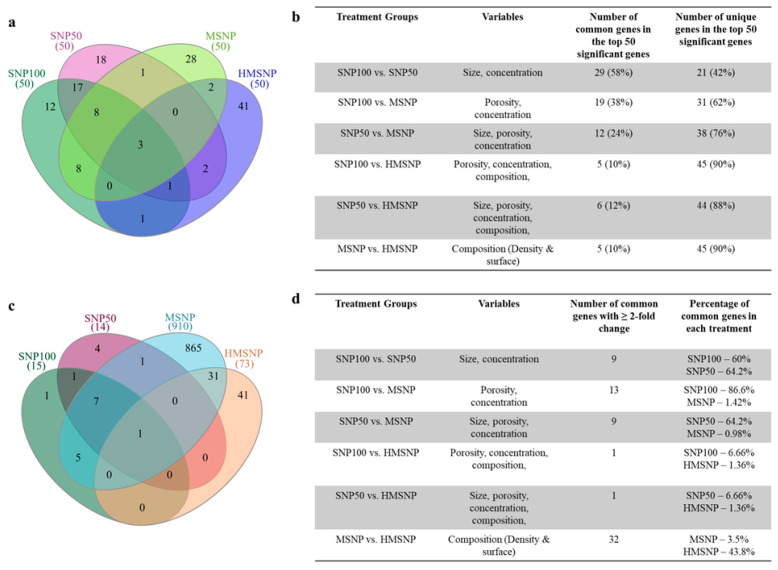
(**a**) The Venn diagram shows the common and unique genes between different treatment groups in their top 50 most significant (based on padj-value) genes. The Venn diagrams were generated using InteractiVenn, a web-based online tool [25]. (**b**) The table shows the number of common and unique genes between different treatment groups in their top 50 most significant genes. (**c**) The Venn diagram shows the common genes between different treatment groups for genes with ≥2-fold change (based on fold change). (**d**) The table shows the number of common genes between different treatment groups for genes with ≥2-fold change.

The changes in gene expression upon SNP saturation were investigated by RNA-sequencing. The DESeq2 package (v1.42.1) was utilized to analyze gene expression, and the apeglm method was used to identify differentially expressed genes among the SNP treatment groups. Figure 2a shows the principal component analysis (PCA) in the four SNP-treated groups compared to the nontreated control group. The non-porous SNP100 and SNP50-treated groups, at their maximum nontoxic doses, did not show significant variance in the PCA analysis among themselves or in comparison with the nontreated controls. In contrast, the MSNP-treated group showed the highest variance in PC1, whereas the HMSNP-treated group showed the highest variance in PC2 compared to the other groups. The close packing of the sample points in the graph showed less variance among the same treatment groups. The volcano plots in Figure 2b–e show the upregulated and downregulated genes in different treatment groups along with their respective statistical significance. The MSNP- and HMSNP-treated groups show the largest number of differentially expressed genes with high statistical significance. The overall gene expression heatmap in Figure 2f shows the relative Euclidean distances between the samples. The blue color in the heatmap indicates a greater Euclidean distance, while red indicates a smaller Euclidean distance among the samples. From the overall heatmap, the MSNP-treated group showed the greatest distinction in gene expression, whereas the others were more similar to each other. The number of upregulated genes (Figure 2g) in the SNP-treated groups was 251, 253, 2670, and 922, and the number of downregulated genes (Figure 2g) was 253, 435, 2339, and 1035 for SNP100, SNP50, MSNP, and HMSNP-treated groups, respectively, compared to the non-treated controls. Genes with more than twofold change in expression were 15, 14, 910, and 73 for SNP100-, SNP50-, MSNP-, and HMSNP-treated groups, respectively, compared to the nontreated controls. The numbers quantitatively confirm the findings with the PCA plot, volcano plot, and overall heatmap, with the highest number of gene expression changes in the MSNP-treated group, the HMSNP-treated group being the second highest, and the lowest number being in the SNP100- and SNP50-treated groups compared to the nontreated control. The top 50 differentially expressed genes for the SNP treatment groups are provided as supplementary information (Supplementary document S1, Figures S1–S4). Among the top 50 most significantly differentially expressed genes, the Venn diagram shows the number of commonly and uniquely expressed genes between the treatment groups (Figure 3a,b). SNP100- and SNP50-treated groups showed the highest number of 29 commonly expressed genes among their top 50 differentially expressed genes. The HMSNP-treated group showed the lowest number of common genes compared to the other treatment groups—specifically, five with SNP100 and the MSNP-treated group and six with the SNP50-treated group. The list of common genes between treatments is mentioned in Table S1 in Supplementary document S1. Three genes, Atf3, Pald1, and Pmp22, were common to all treatment groups. An upset analysis of the upregulated and downregulated genes between each treatment group is provided in Supplementary document S1 (Figures S9 and S10).

### 3.3. Co-Expression Analysis

**Figure 4:**
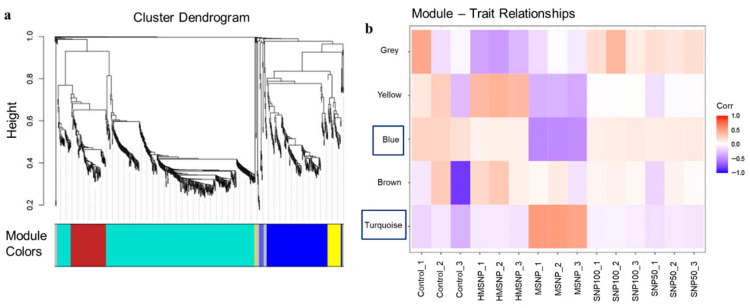
(**a**) Weighted gene co-expression network analysis-derived cluster dendrogram; (**b**) module–trait relationship, which shows the correlation of different treatment groups and gene interaction from a gene-centric approach. Blue and Turquoise modules are highlighted for being the most significant ones.

**Figure 5:**
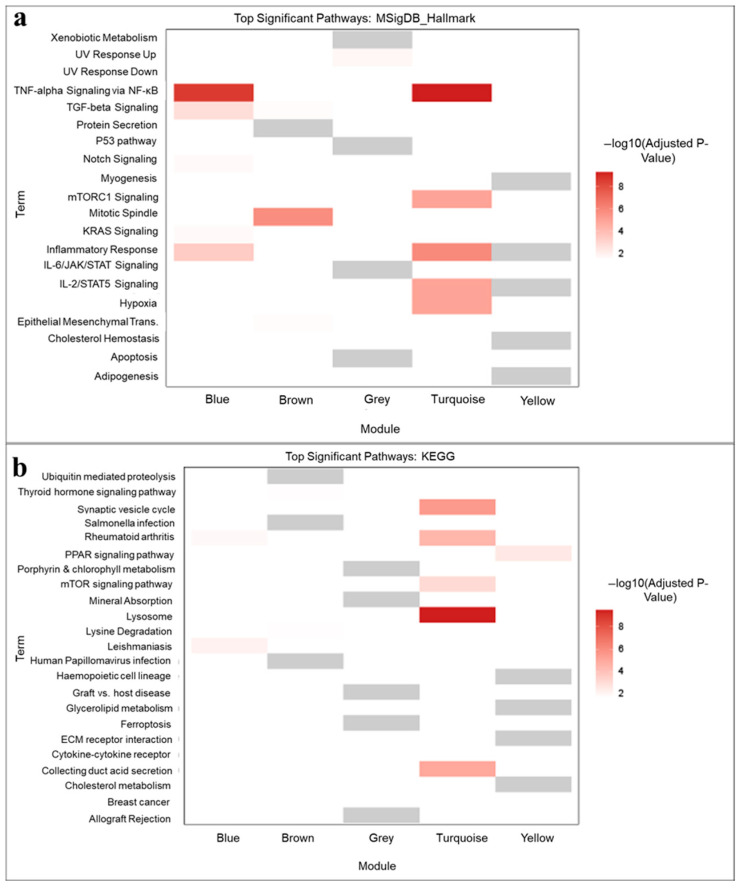
(**a**) Hallmark analyses based on WGCNA showing the correlation with significant Hallmark pathways; (**b**) KEGG analyses based on WGCNA showing the correlation with significant KEGG pathways. The red boxes show the correlation with the modules and respective pathways, as indicated by a significance level of −log10 *p*-value. Grey boxes are not statistically significant.

To check the co-expression of genes upon SNP saturation, we used weighted gene co-expression network analysis (WGCNA). Figure 4a displays the cluster dendrogram and the corresponding color-coded modules, illustrating the hierarchical clustering of genes based on their co-expression similarity. Figure 4b illustrates the individual samples and their correlation with the color-coded modules through the module–SNP relationship. Modules are highly interconnected gene clusters based on correlations. From the cluster dendrogram, the turquoise module is the most significant, followed by the blue module, which is the second most significant, with a lower vertical axis height and highly correlated and co-expressed genes. The module–SNP relationship revealed that the turquoise module exhibits a negative correlation with the control, SNP100-, SNP50-, and HMSNP-treated groups, whereas the MSNP-treated group shows a positive correlation. On the contrary, the blue module showed a positive correlation with the control, SNP100-, SNP50-, and HMSNP-treated groups and a negative correlation with the MSNP-treated group. Checking the related Hallmark pathways in Figure 5a, the turquoise module showed significance in the TNF-alpha via NF-kB pathway, mTORC1 pathway, inflammatory response, and IL-2/STAT5 signaling pathway. This indicates the MSNP-treated group has a positive correlation, while the others have a negative correlation with these pathways. Additionally, the blue module identified TNF-alpha via the NF-kB pathway, the TGF-beta pathway, and the inflammatory response pathway as the top significant pathways. Here, the control, SNP100-, SNP50-, and HMSNP-treated groups are positively correlated, while the MSNP-treated group is negatively correlated. This illustrates the concept that the MSNP-treated group is oppositely correlated with the other groups in terms of co-expression analysis. The turquoise module in the top significant KEGG pathway analysis (Figure 5b) showed a positive correlation between the MSNP-treated group and the mTOR pathway; a similar response was observed in the Hallmark analysis. It also showed a positive correlation with lysosomes in MSNP-treated samples, while the others were negatively correlated. The names of the co-expressed genes in the associated Hallmark and KEGG pathway analyses with significance (*p*-value), adjusted *p*-value, and combined scores are mentioned in Supplementary document S3.

### 3.4. Hallmark and KEGG Pathway Analyses

**Figure 6:**
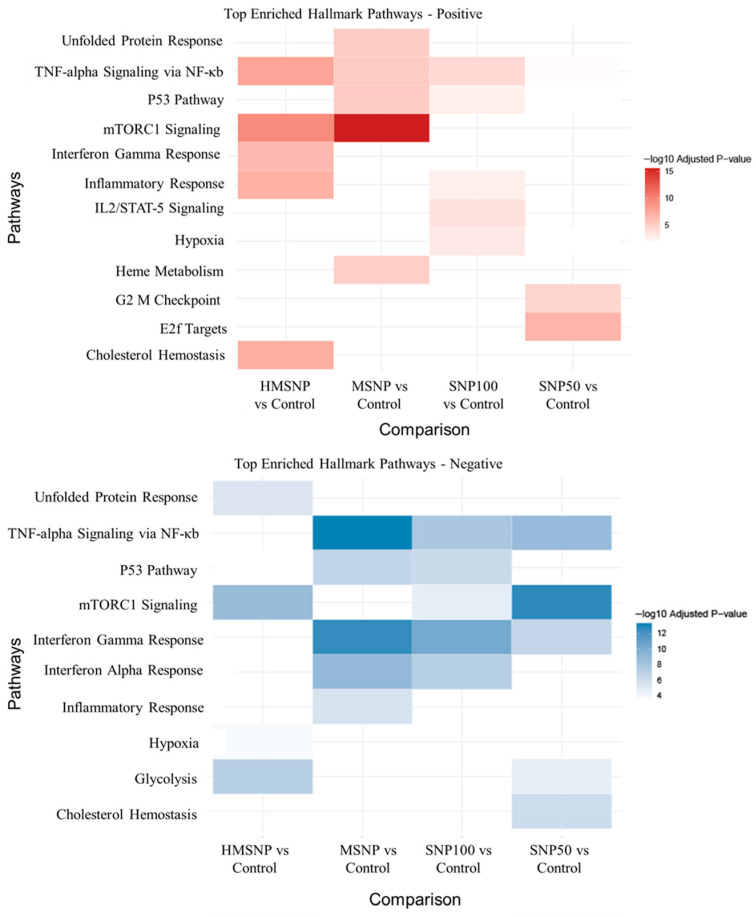
Hallmark pathway analyses show upregulation and downregulation of several immune pathways in the SNP-treated groups compared to nontreated controls. The scales are according to the −log10 adjusted *p*-value.

Molecular Signature Database Hallmark pathway analysis was conducted to identify positively and negatively enriched pathways in the SNP treatment groups (Figure 6). The analysis showed TNF-alpha signaling via the NF-κb pathway in all four SNP-treated groups compared to the control. The MSNP-, SNP100-, and SNP50-treated groups showed both upregulation and downregulation of the pathways; however, the HMSNP-treated group showed only the upregulation of the pathway. Additionally, the p53 pathway was upregulated in SNP100-, SNP50-, and MSNP-treated groups and downregulated in the HMSNP, MSNP, and SNP100 groups. The mTORC1 pathway was upregulated in the porous MSNP and HMSNP groups, but not in the nonporous SNP100 and SNP50 groups, although it was downregulated in the HMSNP, SNP100, and SNP50 groups. The inflammatory response pathway was upregulated in the HMSNP- and SNP100-treated groups and downregulated in the MSNP-treated group. The interferon gamma pathway was upregulated in the HMSNP group and downregulated in all other groups.

KEGG pathway analysis (Supplementary document S1, Figure S11) also showed that TNF-alpha signaling was downregulated in the MSNP-treated group. Also, strong lysosomal upregulation was seen in the MSNP-treated group. Although there are multiple other disease-related pathways, positive and negatively enriched, the focus of this manuscript is particularly on the inflammatory and apoptotic pathways. The enrichment analysis in the Hallmark and KEGG pathways, including the names of the genes, significance values (*p*-values), adjusted *p*-values, and combined scores, is included in Supplementary Document S2.

### 3.5. Th1/Th2 Cytokine Analysis

**Figure 7:**
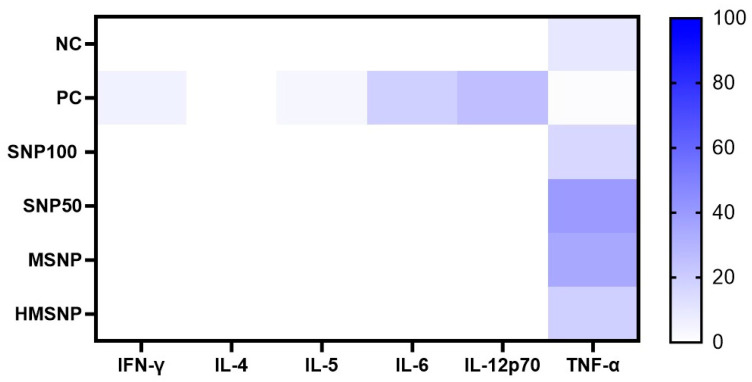
Cell-secreted cytokine responses after 6 h of SNPs treatment via 6-plex Th1/Th2 cytokine panel analysis. Three independent experiments were performed, with three repeats for each sample, and the results were analyzed. Human patient blood serum was used as a positive control to validate the system.

Th1/Th2 cytokine panel analysis was performed (Figure 7) to check the influence of SNP treatment on the M1/M2 polarization of RAW 264.7 cells. IFN-γ, IL-4, IL-5, IL-6, IL-12p70, and TNF-α cytokine levels in the cell culture media upon 6 h of SNP treatment were checked. Except for TNF-α, the levels of the other cytokines were below the lowest background value, which was undetectable in the cell culture media. TNF-α levels showed a significant increase in the TNF-α production in all the treated groups, with 9.36 ± 0.57 pg/mL, 15.82 ± 0.34 pg/mL, 39.92 ± 4.54 pg/mL, 34.69 ± 6.42 pg/mL, and 18.93 ± 0.61 pg/mL for the control, SNP100-, SNP50-, MSNPs-, and HMSNP-treated groups, respectively. The presence of IFN-γ, IL-5, IL-6, and IL-12p70 in the positive control confirmed the system’s validity.

## 4. Discussion

There is a lack of in-depth research on immune cell saturation by nanocarriers for drug delivery applications. In our recently published study, we saturated RAW 264.7 macrophages with SNPs of varying physicochemical properties (the same SNPs used in this study) to observe the effects of size, porosity, density, and composition on the survival and functions of the saturated macrophages [18]. We demonstrated that at non-toxic concentrations, SNPs do not induce any visible effects on apoptosis/necrosis, cell cycle phase distribution, or membrane integrity, as assessed using functional biological assays [18]. We also observed that the SNPs increased the CD80 M1-type surface marker in all treatment groups compared to non-treated controls over time, without interfering with the cells’ phagocytic activity [18]. For the HMSNP-treated cells, this increase in phagocytic activity was evident upon SNP saturation [18]. Therefore, in the current study, we aimed to investigate molecular-level changes in macrophages induced by SNP saturation as a function of physicochemical properties.

**Figure 8:**
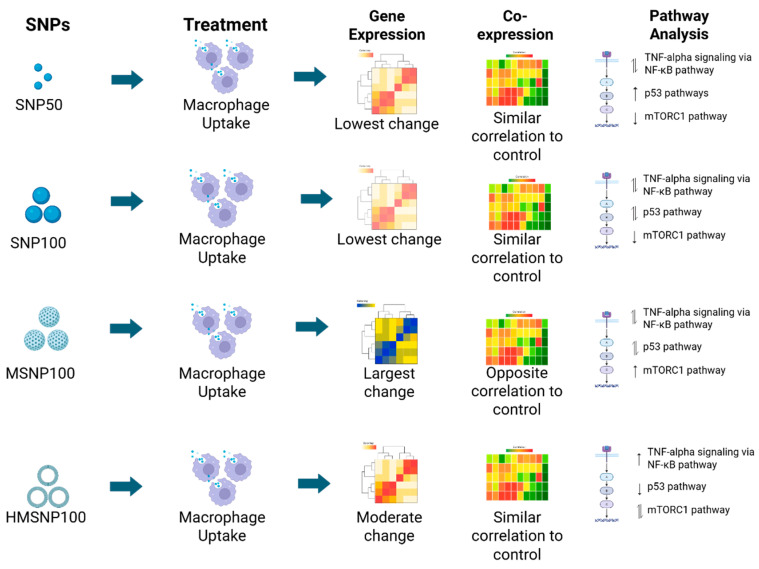
Schematic showing the SNP treatments and their corresponding changes in the gene expression level, co-expression of genes, and associated pathways. The heatmaps and pathway figures in the schematic show the main concepts only and were created in BioRender. Saha, S. (2025) https://BioRender.com/6uoytbc (accessed on 3 December 2025).

We examined the global gene expression changes following SNP treatment at 6 h of saturation time (Figure 8). Although gene expression levels can change significantly depending on incubation time, this study focused on the early changes in gene expression levels upon SNP saturation. The PCA plot, volcano plots, overall gene expression heatmap, and number of upregulated and downregulated genes indicated that SNP100- and SNP50-treated groups have a similar overall effect on the cells, with 15 and 14 genes identified as having more than a twofold change, respectively. We analyzed the common and unique genes in different groups using two approaches: the top 50 differentially expressed genes with the highest significance level and the genes that showed a change of twofold or greater compared to the nontreated controls, since genes with less than a twofold change might not correlate with physiological significance [26]. SNP100- and SNP50-treated groups also showed that 58% of their top 50 significant differentially expressed genes were commonly expressed. The remaining 42% of the unique genes expressed resulted from the effects of the two variables, size and nanoparticle concentration. The effect of concentration is larger than size, as previous studies have also shown that there were minimal changes in the gene expression profile due to nanoparticle size at a similar concentration [20]. For the genes with more than a twofold change, the SNP100- and SNP50-treated groups showed the highest similarity, with 9 common genes out of 15 and 14 common genes for the SNP100- and SNP50-treated groups, respectively. This also validates that nanoparticle size had a minimal impact on gene expression. Between the SNP100- and MSNP-treated groups, 19 common genes (38%) and 31 unique genes (62%) were found in their top 50 differentially expressed genes. Since the size of these SNPs was similar, the difference in gene expression resulted from changes in porosity and the nanoparticle concentration used. Additionally, there are 13 common genes with twofold or greater change in both groups (13 out of 15 for SNP100, and 13 out of 910 for MSNP). A previous study showed that, at a similar concentration and incubation time, nonporous Stober 500 nm and mesoporous 500 nm SNPs share 63–70% of commonly expressed genes, with twofold changes or more [19]. This indicates that there are some common genes being differentially expressed in any SNP-treatment, just as in a foreign nanoparticle administration. However, in the case of porous nanoparticles and increased administered concentration, the number of differentially expressed genes increases. As a result, at an equitoxic concentration and incubation time (MSNPs have a higher equitoxic concentration than SNP100), both porosity and concentration contributed to the change in gene expression. The SNP50- and MSNP-treated groups shared 12 common genes (24%) in the top 50 genes, which is fewer than the SNP100- vs. MSNP-treated groups (38%), as these two groups differ in three parameters: size, porosity, and concentration. This supports the notion that size contributes to the change in gene expression; however, it contributes less compared to porosity and concentration. The HMSNP-treated group showed only 5–6 (10–12%) genes in common with the other treatment groups, indicating the profound impact of nanoparticle composition (density and surface composition) in the top 50 differentially expressed genes. SNP50 and SNP100 had a lower equitoxic concentration, indicating a lower number of nanoparticles administered compared to MSNP- and HMSNP-treated cells, which had four to eight times higher equitoxic concentrations involving a higher number of nanoparticles in the system. A higher number of nanoparticles indicates more cell–nanoparticle interactions and hence a higher gene expression alteration based on the results observed. As for the non-toxic concentrations for MSNPs and HMSNPs, although equal, HMSNPs have a very low density and a distinct surface composition, which could potentially slow nanoparticle uptake in cells and result in lower cell–nanoparticle interactions than the MSNPs. This could result in a relatively lower number of differentially expressed genes and genes with a twofold change in the HMSNP-treated group compared to the MSNP-treated group. However, the overlap analysis plots (Supplementary document S1, Figures S9 and S10) in MSNP- and HMSNP-treated groups show very significant overlaps (1272 upregulated genes and 1656 downregulated genes) in both the treatment groups, which shows that a similar porous surface, size, and concentration contributed to the overlaps in these two treatments. The HMSNP-treated group showed more unique genes expressed compared to SNP100-, SNP50-, and MSNP-treated groups in the top 50 genes, which also indicated that surface compositions can significantly dominate the gene expression changes and associated pathways more than size and porosity. A recent study showed that surface-modified gold nanoparticles differentially expressed genes via functional annotations and weighted gene correlation network analysis in two different human cell lines [27]. Surface modification techniques, including surface charge, surface coating, and composition, dictate protein binding, electrostatic interactions with the cell membrane, and the cellular uptake rate, thereby affecting cell–nanoparticle interactions and corresponding gene expression changes [27], as observed here also.

As mentioned in the results, three genes among the top 50 differentially expressed genes were common in all the treatment groups. Activating transcription factor 3, also known as Atf3, functions as a hub of adaptive responses in cells, which is also linked to multiple immune signaling pathways, including NF-κB, p53, JNK, etc. [28]. An upregulation of Atf3 was found in all the treatment groups, which indicates a stress-induced cellular behavior and prevention of the system from immune overreaction. Additionally, the Pmp22 gene, which is responsible for a complex mechanism regulating the actin skeleton, cell adhesion, migration, and growth, was found to be upregulated in all treatment groups compared to controls [29]. This indicates the involvement of a gene related to the phagocytic uptake of nanoparticles by macrophages, facilitated by integrins and actin cytoskeleton remodeling [30,31]. The third gene, Pald1, is responsible for encoding paladin, a membrane protein responsible for endosomal trafficking and VEGFR2 internalization [32]. Though their behavior in macrophages has not been studied extensively, its expression might indicate the dynamics involved in SNP internalization in the cells compared to the nontreated cells.

We focused on the pathways that are associated with the immunotoxicity of macrophages following SNP exposure. Using a gene-centric approach, WGCNA was first employed to identify co-expressed genes contributing to biological processes and toxicity pathways. Additionally, database resources such as Hallmark (MSigDB) and KEGG pathway analysis were utilized to corroborate the findings of the WGCNA analysis. The top significant Hallmark pathways from co-expression analysis showed the pro-inflammatory/stress-response (TNF-alpha via NF-kB pathway, TGF-beta signaling) [33,34], metabolic/cell growth (mTORC1 pathway) [35], inflammatory pathway (inflammatory response), and cytokine signaling–immune activation (IL-2/STAT5 pathway) [36] as the top significant pathways. As discussed in the results, the MSNP-treated group correlates oppositely with the other treatment groups, and SNP100-, SNP50-, and HMSNP-treated groups show a similar correlation to the nontreated controls. However, all the groups differ in their significance level, which also indicates the physicochemical property-dependent cellular response.

TNF-alpha signaling influenced by SNP exposure was previously reported in the literature, and it is common as a pro-inflammatory and stress response mechanism upon nanoparticle exposure [19,37]. Additionally, it is also a strong indication for macrophages with M1-type polarization behavior. Previously, we observed increased CD80 expression, a marker for M1-type polarization in response to SNPs, which confirms the involvement of TNF-alpha via the NF-kB pathway [18]. However, the negative correlation of TNF-alpha via the NF-kB pathway also indicates a set of genes regulating the pathway. The literature showed the complex behavior of the NF-kB pathway and the self-regulating behavior in macrophages [38,39]. Using a predefined supervised database resource, Hallmark and KEGG pathway analyses were employed to explore the SNP-induced macrophage behavior upon saturation. First, upregulation of TNF-alpha signaling via the NF-kB pathway was observed in all the treatment groups, and downregulation of the pathway was also observed in all the groups except the HMSNP-treated group, which largely corroborates the finding from the WGCNA analysis.

The MSNP-treated group also showed a positive mTOR1 correlation in WGCNA analysis, indicating increased cell growth and proliferation in the treatment, which was also noticed in previous studies involving mesoporous silica nanoparticles [40,41]. Hallmark pathway analysis also confirmed that the mTORC1 signaling pathway was upregulated in the MSNP-treated group and the HMSNP-treated group and downregulated in the HMSNP-, SNP100-, and SNP50-treated groups. This indicates SNP100 and SNP50-treated groups tend to conserve energy, recycling, and repair, which might not promote growth, while the MSNP-treated group promotes cell growth and metabolism [35]. The KEGG pathway analysis (Figure 5b) revealed significant lysosomal activation in the MSNP-treated group, which was also reported before in MSNP-treated macrophages [20]. Previous reports also showed that the lysosomal upregulation of the MSNP-treated group is connected with mTORC1 upregulation since lysosomal acidification due to degradable MSNPs can activate mTOR serine/threonine protein kinase, leading to the activation of the mTORC1 signaling pathway [41]. The HMSNP-treated group showed both upregulation and downregulation of the pathway, indicating a more controlled cell growth and metabolism behavior via a feedback mechanism. A reason might be the lower density of HMSNPs compared to MSNP, which possibly indicates that a longer incubation time with HMSNP might also show similar behavior to the MSNP-treated group. Overall, the modulation of the mTORC1 pathway suggests that porous SNPs tend to upregulate the pathway while promoting growth, whereas non-porous SNPs do not exhibit this behavior.

The inflammatory response Hallmark pathway is a broad immune response that includes all the pathways involved in the different treatment groups. Additionally, downregulation of the interferon gamma pathway in the same groups also confirms the regulation of M1-type proinflammatory behavior. Though RAW 264.7 cells do not secrete interferon gamma, downregulation of the interferon gamma pathway signifies the downregulation of genes that are also involved in interferon gamma signaling pathways [36]. Another important pathway involved in the analysis was the p53 pathway, which also has a broad range of functions known as the guardian of the genome stress pathway [42]. The HMSNP-treated group showed downregulation, and the SNP50-treated group showed upregulation; the MSNP and SNP100-treated groups showed both up- and downregulation. Upregulation of the p53 pathway is closely related to the stress response and repair mechanism in the cells caused by nanoparticle uptake [43]. The SNP50-treated group indicates an upregulation of the pathway, which indicates the stress response, damage, and repair mechanisms in the cells, consistent with previous observations that SNP50 showed higher cytotoxicity at a lower maximum nontoxic concentration [18], which may correlate with p53 pathway activation. Both up- and downregulation of the p53 pathway in the mesoporous and SNP100-treated groups indicate an initial stress and damage response, which is quickly followed by a resolution mechanism.

The KEGG pathway analysis showed pathways involved in the disease mechanism, which was not a focus of this particular study. The key finding from KEGG pathway analyses was the upregulation of lysosomal pathways in the MSNP-treated group. Gene ontology analyses (Supplementary Figures S12 and S13) for biological processes also showed a strong upregulation in vesicle-mediated transport (GO:0016192), lytic vacuole organization (GO:0080171), and lysosomal transport (GO:0007041) processes, indicating increased lysosomal stress, trafficking, and endocytosis in the MSNP-treated group, leading to the lysosomal activation pathway.

On the whole, the four SNP-treated groups showed a complex macrophage signaling response based on their physicochemical properties at equitoxic doses. SNP100- and SNP50-treated groups show similar responses with TNF-alpha via NF-kb up and downregulation, interferon gamma downregulation, and mTORC1 signaling downregulation for both groups, which suggests that both the SNPs show a pro-inflammatory response with a negative regulatory feedback mechanism, as well as suppressed cell growth and metabolism. The p53 pathway up- and downregulation for SNP100, and only upregulation for the SNP50-treated group, suggest that the macrophages have more damage or more of a stress response upon SNP50 than SNP100, which also strengthens the idea that smaller nanoparticles have a more toxic response. Mixed TNF-alpha signaling via the NF-kb and p53 pathways, with a strong mTORC1 pathway upregulation, suggests the MSNP-treated group promotes the regulation of inflammation while promoting cellular growth, indicating the particle might pose a risk towards oncogenic transformation. The HMSNP-treated group suggests an immunostimulatory effect with a mixed mTORC1 response, with TNF-alpha via NF-kb upregulation and p53 pathway downregulation.

The Th1/Th2 cytokine panel analysis from the cell culture supernatant showed mostly increased TNF-alpha secretion upon treatment compared to the nontreated control, which suggests a proinflammatory response common for most nanoparticle treatments. M1 polarized macrophages also secrete high levels of IL-6 and IL-12 [44,45]. However, the absence of IL-6 and IL-12p70 in the treatment groups suggests only acute pro-inflammatory behavior without strong systemic inflammatory activation and a more controlled response. Usually, RAW 264.7 cells do not produce IL-4, IL-5, and IFN-γ, which were visible in the cytokine panels and validate the results from the cytokine analysis. The overall Th1/Th2 cytokine panel analysis also corroborates the pathway analysis, showing mostly a regulated M1 macrophage response, not involving severe inflammation upregulation or associated cellular damage. This study highlights the early molecular-level immune response of macrophages upon SNP saturation. Time-dependent changes and protein level fluctuations warrant further exploration in future studies.

## 5. Conclusions

The major findings of this study showed that gene expression alterations in SNP-saturated macrophages are mostly due to changes in nanoparticle porosity and surface composition rather than size. The overall pathway analyses showed a feedback mechanism regulating the TNF-alpha via NF-kB signaling, mTORC1 signaling, and p53 pathways in the SNP-treated macrophages. The Th1/Th2 cytokine panel analyses showed that all SNPs at their equitoxic saturation concentrations did not affect cytokine levels, except for a TNF-alpha increase, indicating an acute proinflammatory response. The differences among the treatment groups indicate the need to optimize the dose, duration, and physicochemical properties of the SNPs for specific systemic delivery applications.

## Data Availability

The data are deposited in Gene Expression Omnibus [Gene Expression Omnibus] [https://www.ncbi.nlm.nih.gov/geo/query/acc.cgi?acc=GSE312613] (accessed on 3 December 2025) [GSE312613].

## Supplementary Materials

The following supporting information can be downloaded at: https://www.mdpi.com/article/10.3390/pharmaceutics18030344/s1, Supplementary Document S1: Figure S1: Top 50 gene expression heatmap SNP100 vs. control; Figure S2: Top 50 gene expression heatmap SNP50 vs. control; Figure S3: Top 50 gene expression heatmap MSNP vs. control; Figure S4: Top 50 gene expression heatmap HMSNP vs. control; Table S1: Common genes between treatments in their top 50 significant differentially expressed genes; Table S2: Top 15 genes in each treatment group with 2-fold change; Figure S5. Volcano plot showing the significant differentially expressed genes in SNP100-treated groups vs. control; Figure S6. Volcano plot showing the significant differentially expressed genes in SNP50-treated groups vs. control; Figure S7. Volcano plot showing the significant differentially expressed genes in SNP50-treated groups vs. control; Figure S8. Volcano plot showing the significant differentially expressed genes in SNP50-treated groups vs. control; Figure S9. Overlap analysis of upregulated genes in different treatment groups; Figure S10. Overlap analysis of downregulated genes in different treatment groups; Figure S11. KEGG pathway analyses show upregulation and downregulation of several immune pathways in the SNP treatment group compared to nontreated controls. The scales are according to the −log10 adjusted *p*-value; Figure S12. Gene ontology- biological process analyses show upregulation of biological processes in the SNP treatment group compared to nontreated controls. The scales are according to the -log10 adjusted *p*-value; Figure S13. Gene ontology- biological process analyses show downregulation of biological processes in the SNP treatment group compared to nontreated controls. The scales are according to the −log10 adjusted *p*-value. Figure S14. TNF-α levels in the SNP-treated macrophages detected by ELISA. Supplementary Document S2: Hallmark and KEGG pathway analyses; Supplementary Document S3: Co-expression analyses.
