# Supplementary material for: Modulation of Cell Signaling Pathways in Silica Nanoparticle-Saturated Macrophages
Source: Pharmaceutics. 2026 Mar 11;18(3):344. doi: 10.3390/pharmaceutics18030344 (PMC13030532; doi:10.3390/pharmaceutics18030344)
Supplement: Supplementary file 1 [file pharmaceutics-18-00344-s001.zip › Supplementary document S1.pdf]

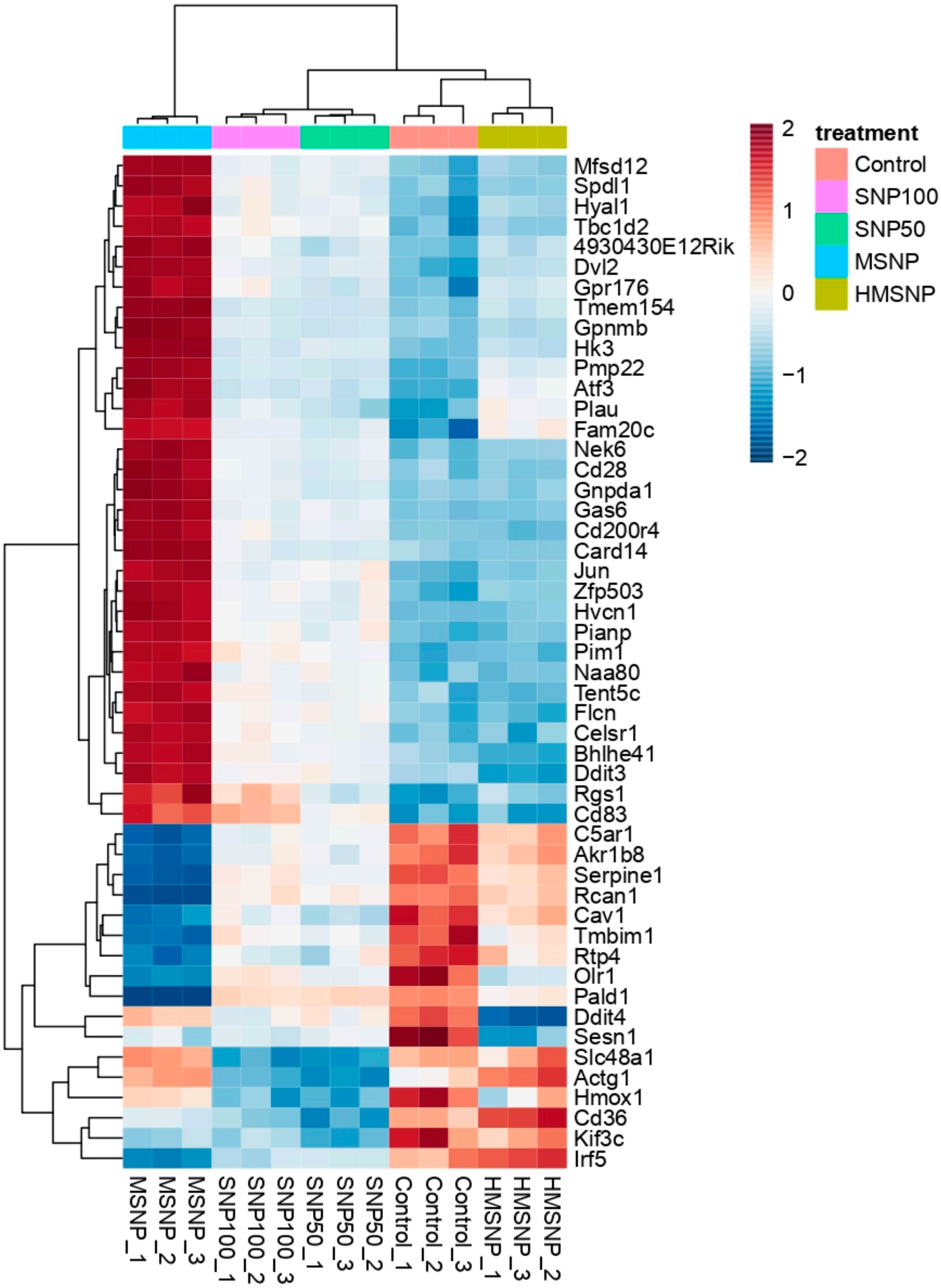

Fig. S1. Top 50 gene expression heatmap SNP100 vs. control

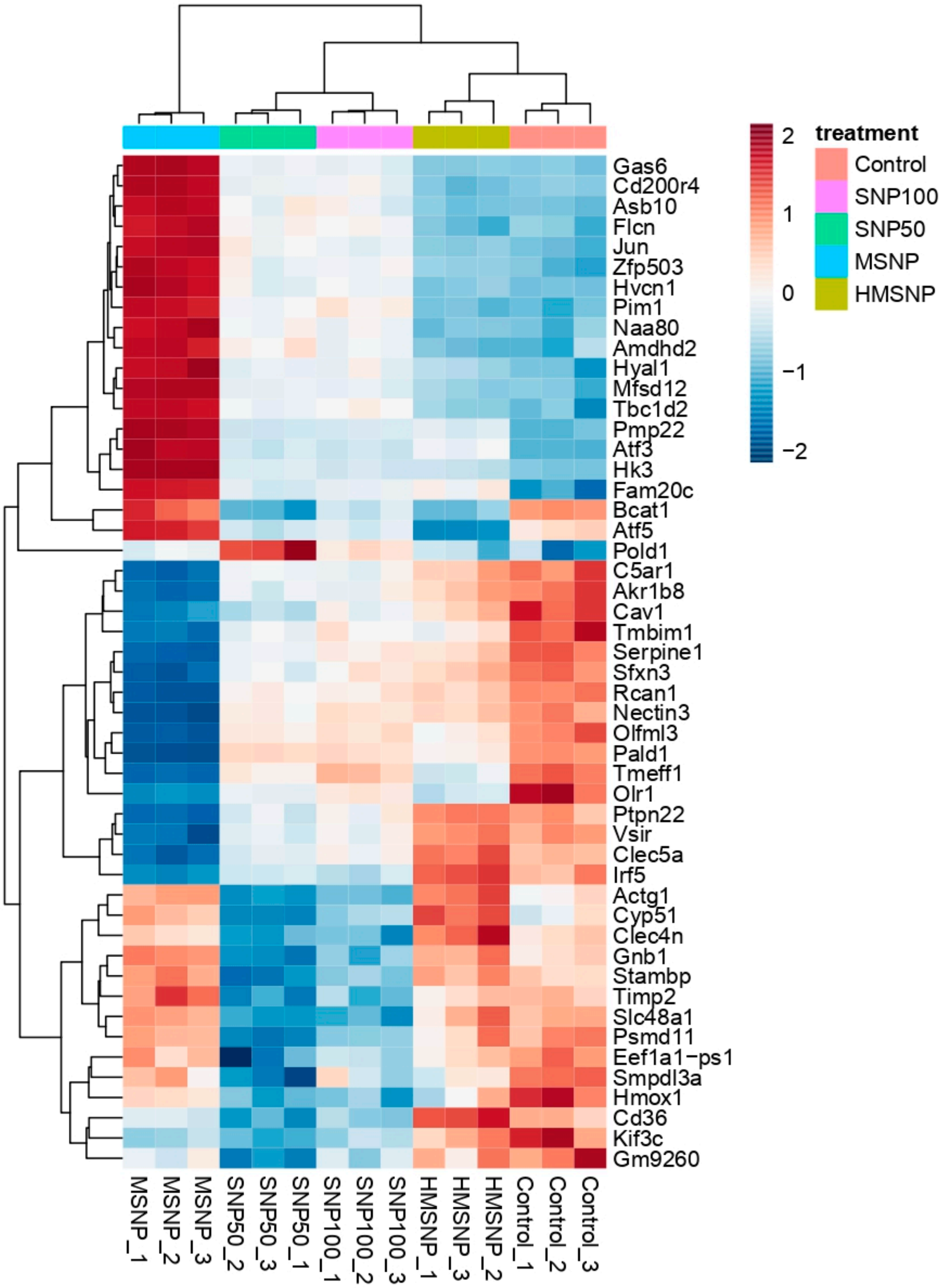

Fig. S2. Top 50 gene expression heatmap SNP50 vs. control

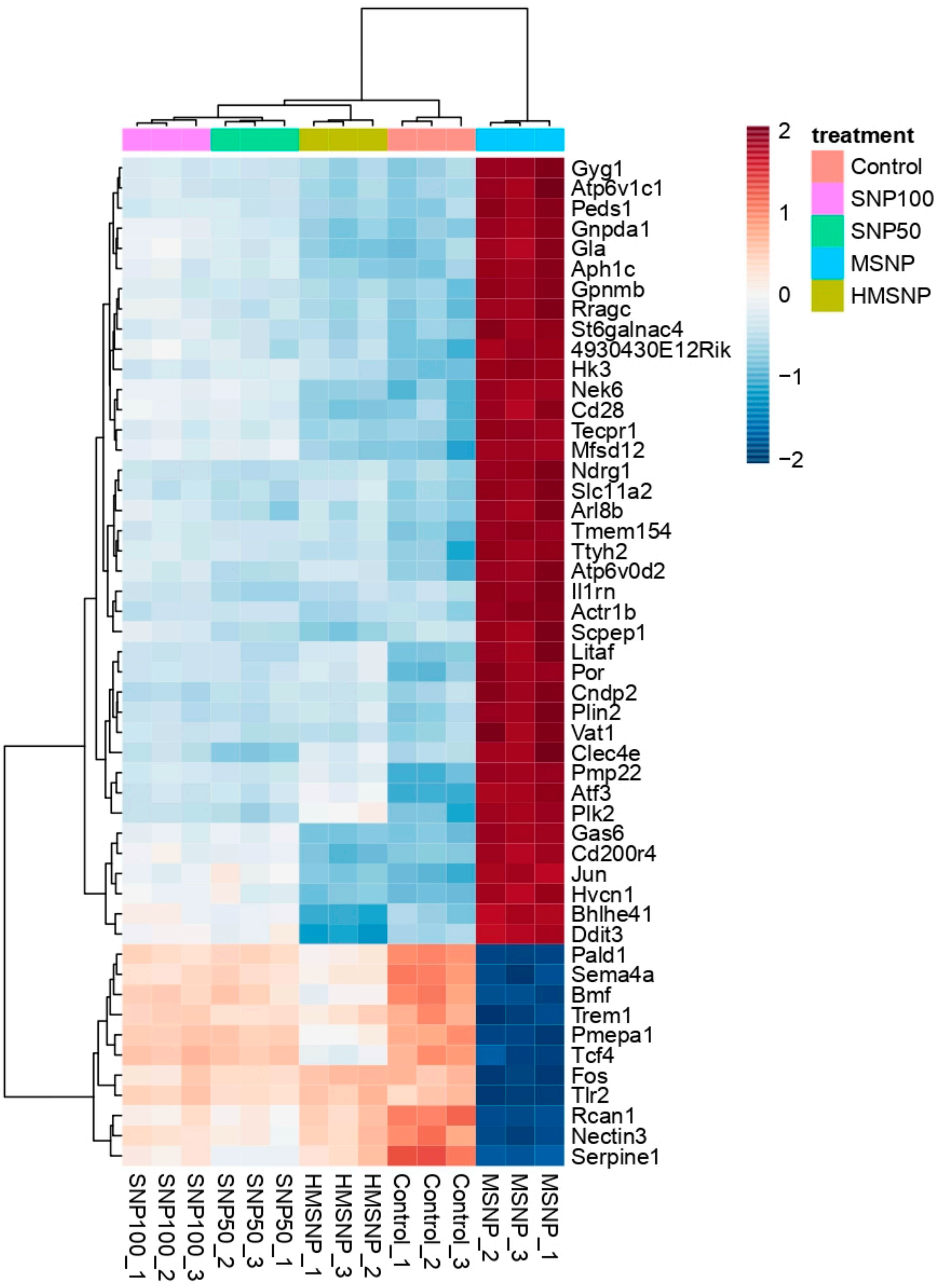

Fig. S3. Top 50 gene expression heatmap MSNP vs. control

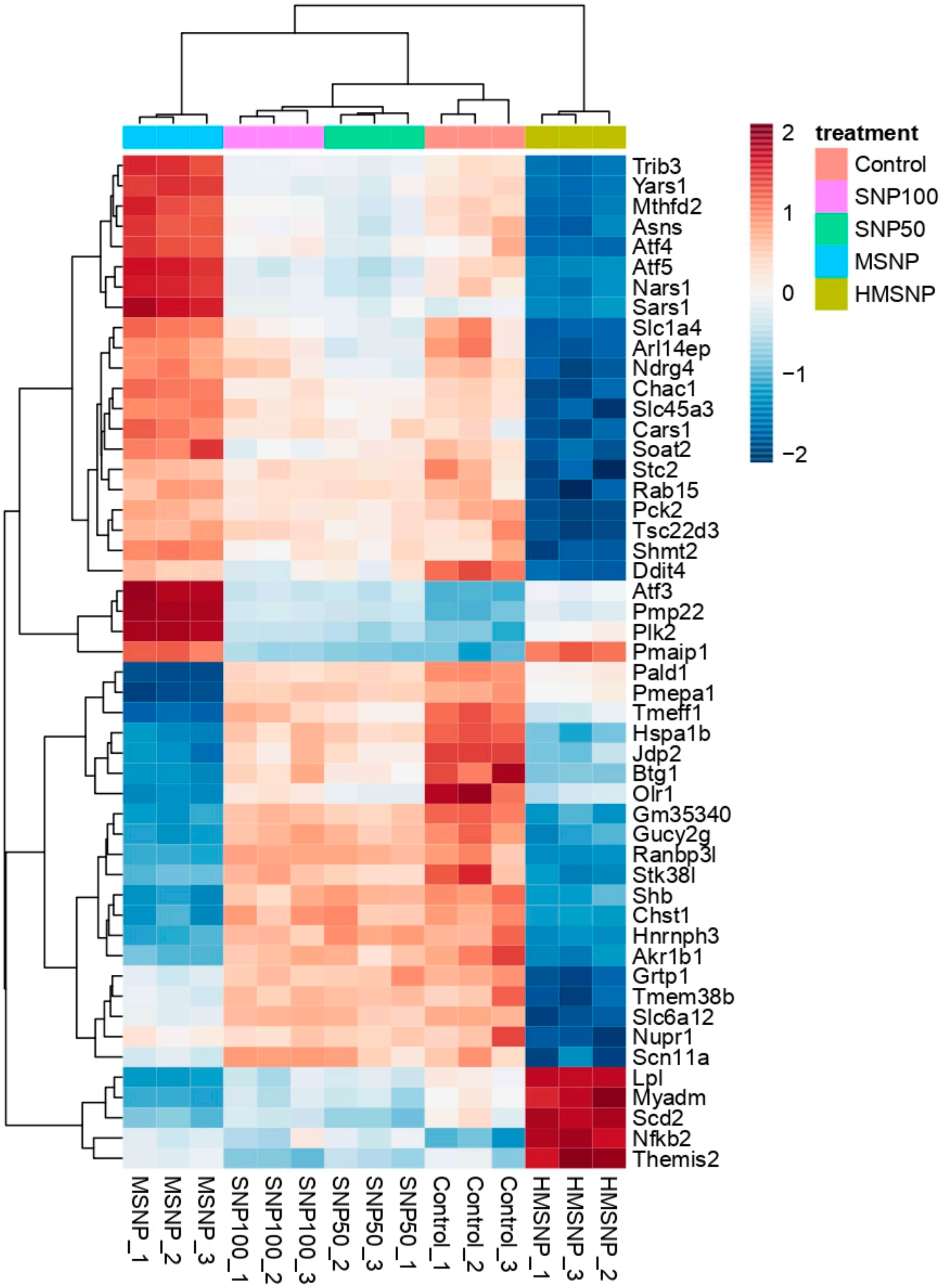

Fig. S4. Top 50 gene expression heatmap HMSNP vs. control

SNP100 vs. SNP50	SNP100 vs. MSNP	SNP50 vs. MSNP	SNP100 vs. HMSNP	SNP50 vs. HMSNP	MSNP vs. HMSNP
Actg1	4930430E12Rik	Atf3	Atf3	Atf3	Atf3
Akr1b8	Atf3	Cd200r4	Ddit4	Atf5	Pald1
Atf3	Bhlhe41	Gas6	Olr1	Olr1	Plk2
C5ar1	Cd200r4	Hk3	Pald1	Pald1	Pmepa1
Cav1	Cd28	Hvcn1	Pmp22	Pmp22	Pmp22
Cd200r4	Ddit3	Jun		Tmeff1	
Cd36	Gas6	Mfsd12			
Fam20c	Gnpda1	Nectin3			
Flcn	Gpnmb	Pald1			
Gas6	Hk3	Pmp22			
Hk3	Hvcn1	Rcan1			
Hmox1	Jun	Serpine1			
Hvcn1	Mfsd12				
Hyal1	Nek6				
Irf5	Pald1				
Jun	Pmp22				
Kif3c	Rcan1				
Mfsd12	Serpine1				
Naa80	Tmem154				
Olr1					
Pald1					
Pim1					
Pmp22					
Rcan1					
Serpine1					
Slc48a1					
Tbc1d2					
Tmbim1					
Zfp503					

Table S1. Common genes between treatments in their top 50 significant differentially expressed genes

SNP100 vs. Control		SNP50 vs. Control		MSNP vs. Control		HMSNP vs. Control	
Genes	Log2fold Change	Genes	Log2fold Change	Genes	Log2fold Change	Genes	Log2fold Change
Tent5c	1.283263	Gm56769	1.18992	Dio2	5.291112	Nupr1	- 1.5239985
Rragd	1.246674	Fabp4	1.110516	Tspan10	4.95755	H1f3	-1.526316
Card14	1.238021	Eqtn	1.059394	Rragd	4.911438	Pck2	- 1.5670620
Celsr1	1.226986	Tent5c	1.050068	Card14	4.681155	H1f4	-1.635167
4833415N18 Rik	1.199035	Hvcn1	1.022393	Il7r	4.285535	H1f5	-1.639790
Gpr176	1.157078	Gm9260	-1.01352	Ighg2c	4.26617	Gucy2g	- 1.6426320
Hvcn1	1.081681	Rsad2	-1.02663	Fabp4	4.263499	Thsd4	-1.644431
H2ac7	1.066829	Rtp4	-1.03102	Efcab6	4.181322	Rab15	-1.751657
Fabp4	1.018412	Serpinb9	-1.13692	Dok7	4.072243	Chst1	-1.909623
Cav1	-1.04349	Hmox1	-1.3609	Sstr5	3.997186	Olr1	-1.979680
Rtp4	-1.0761	Serpine1	-1.37811	4833415N 18Rik	3.926345	Slc1a4	-2.332961
Serpine1	-1.09135	Cav1	-1.4191	Ptpn14	3.8656465	Prss35	-2.450934
Tgfbi	-1.16948	Tgfbi	-1.42727	Rgs16	-3.94939	Trib3	-2.547817
Hmox1	-1.31452	Olr1	-1.72162	Pmepa1	-3.85535	Chac1	-2.652797
Olr1	-1.32922			Bmf	-3.73990	Ranbp3l	-2.785718

Table S2. Top 15 genes in each treatment group with 2-fold change

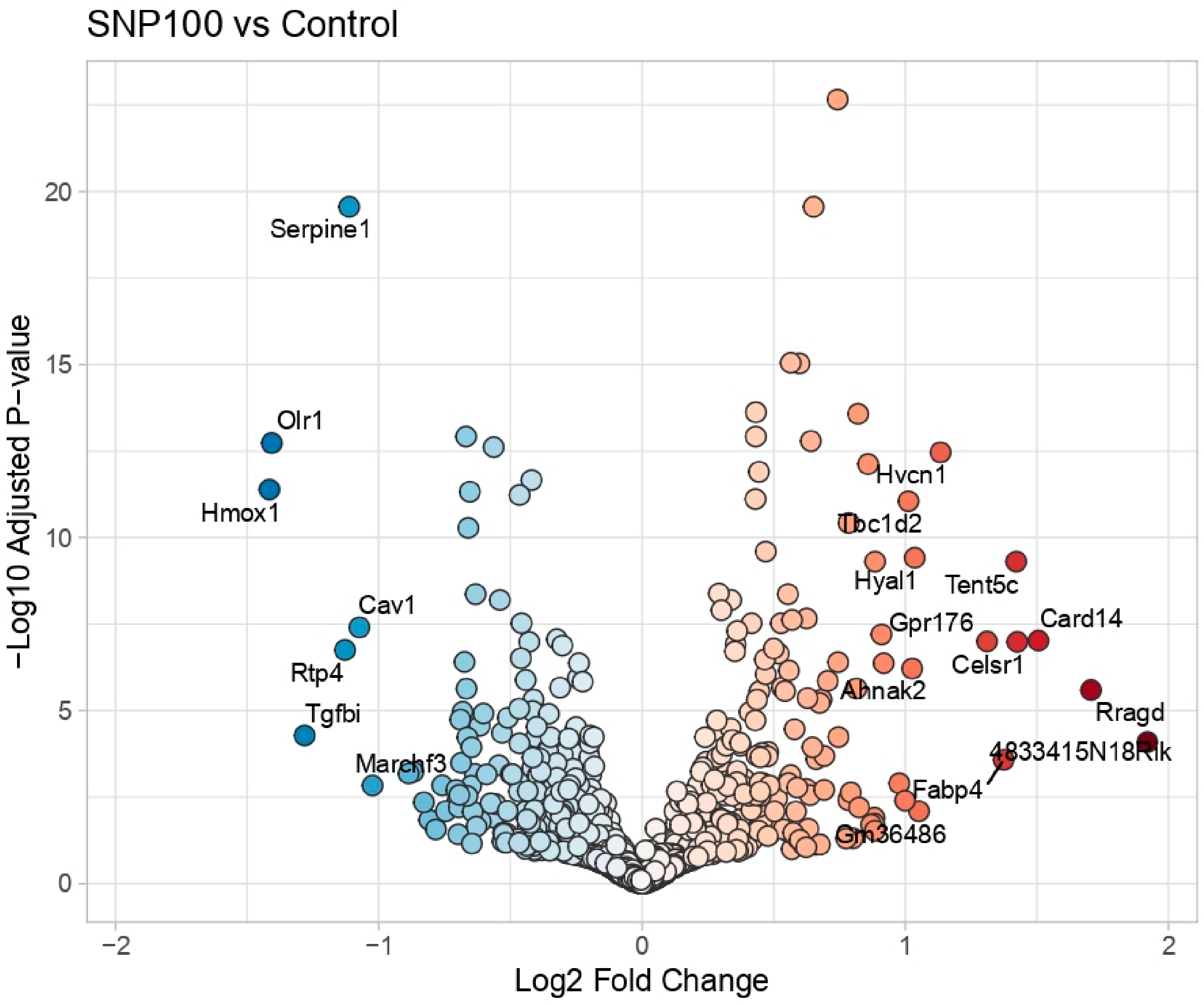

Fig. S5. Volcano plot showing the significant differentially expressed genes in SNP100-treated groups vs. control

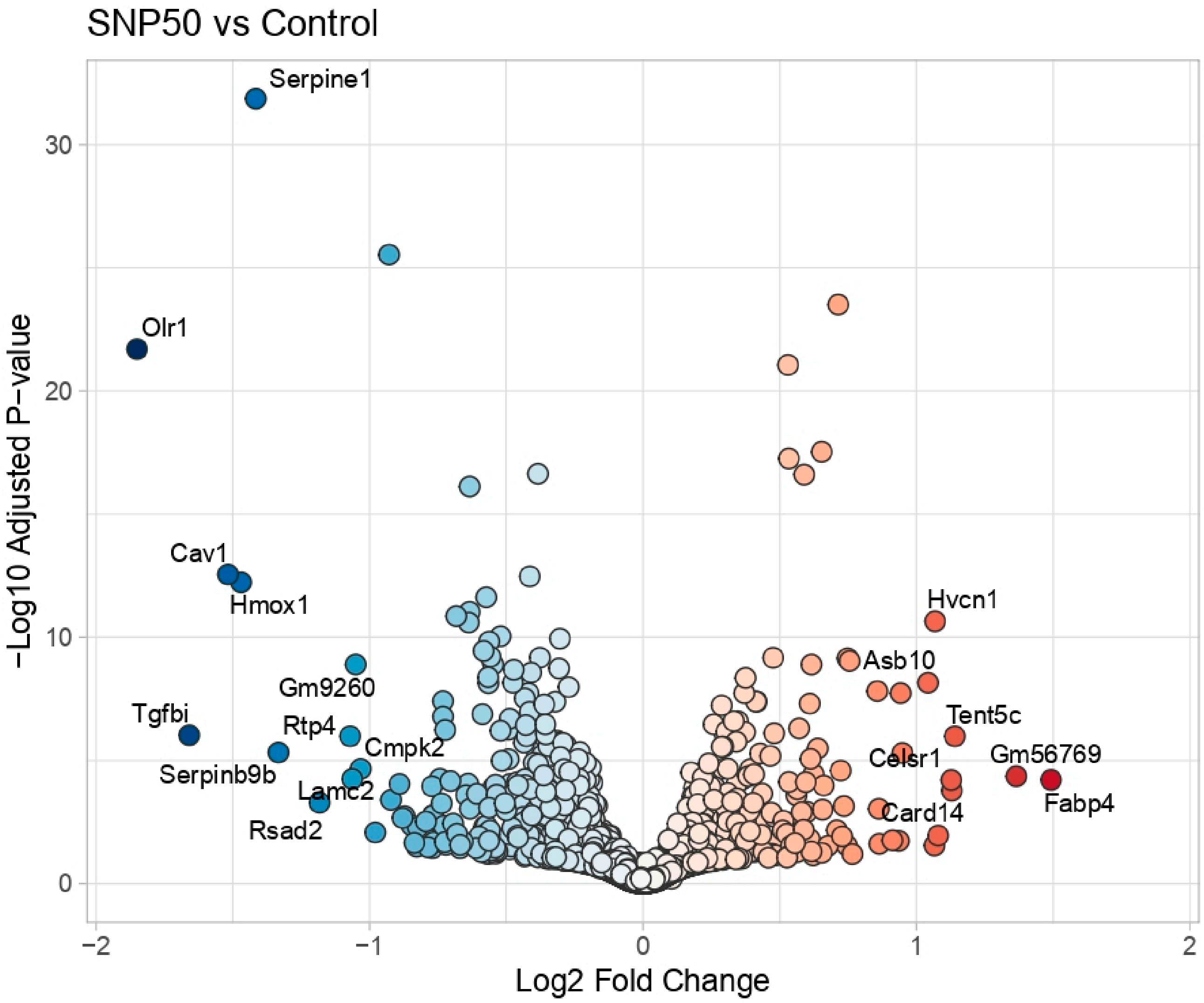

Fig. S6. Volcano plot showing the significant differentially expressed genes in SNP50-treated groups vs. control

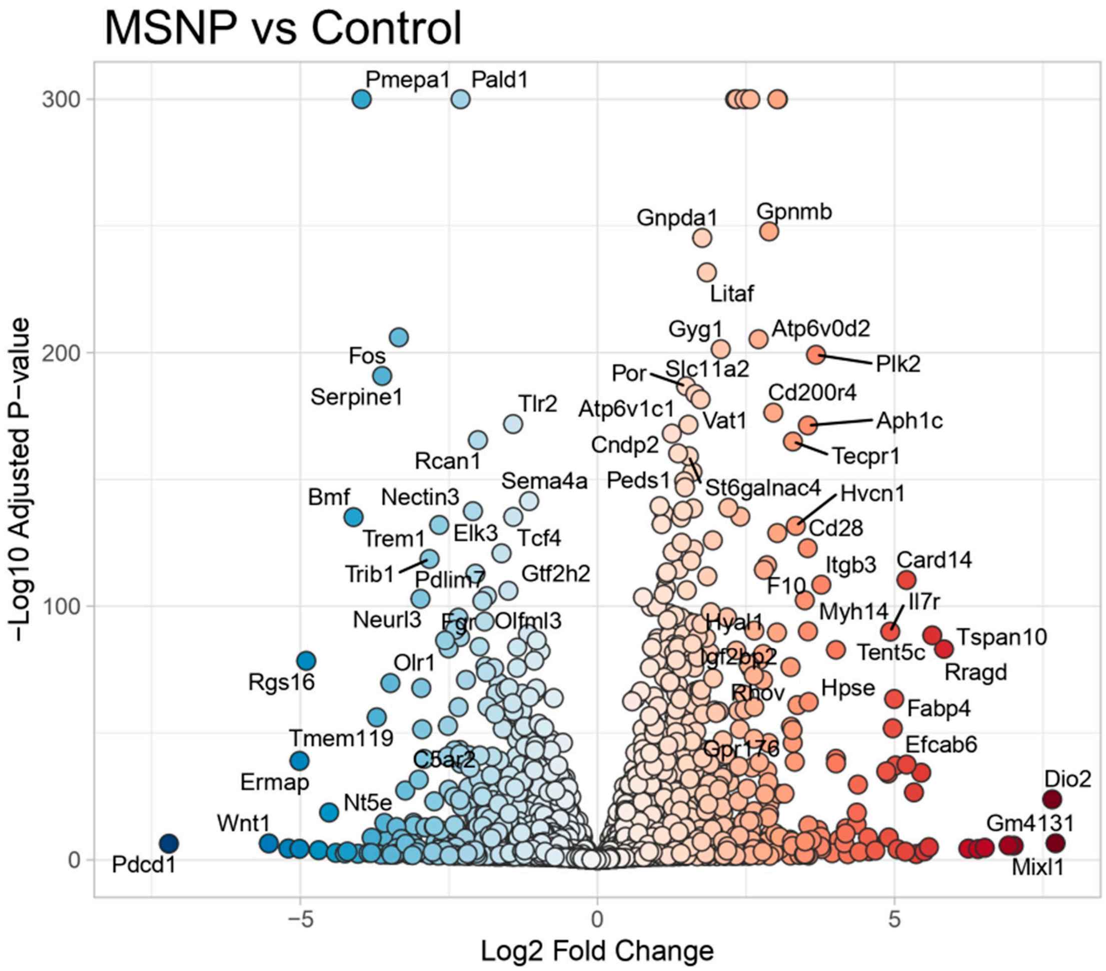

Fig. S7. Volcano plot showing the significant differentially expressed genes in SNP50-treated groups vs. control

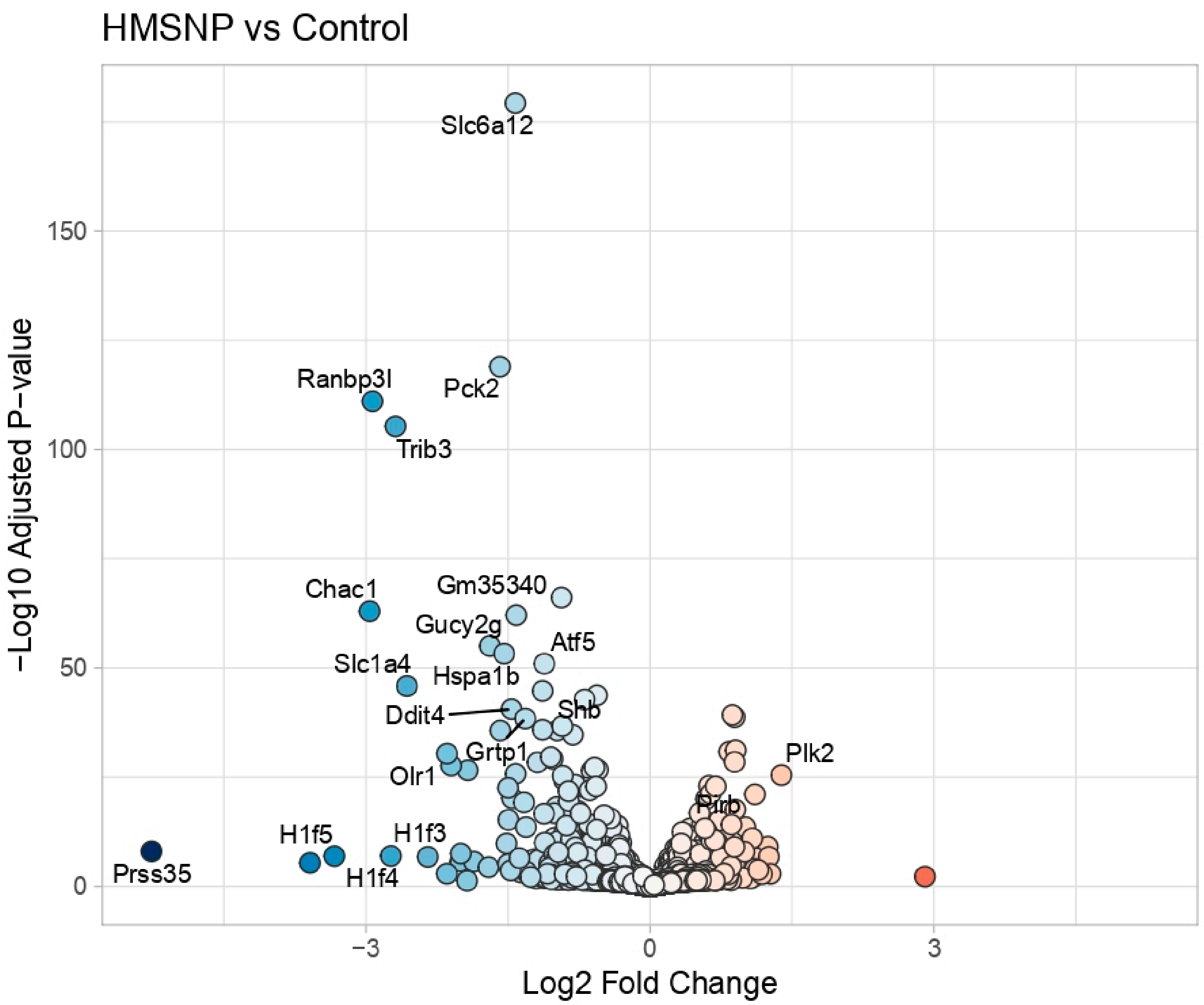

Fig. S8. Volcano plot showing the significant differentially expressed genes in SNP50-treated groups vs. control

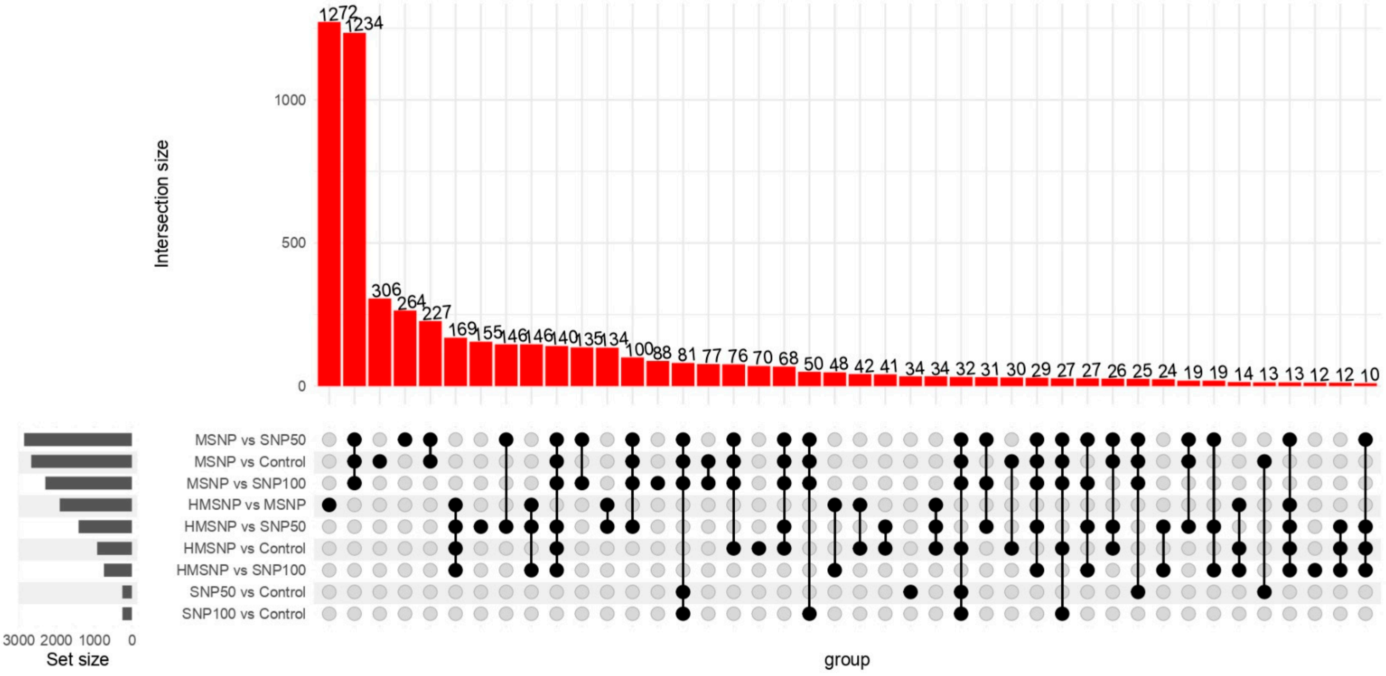

Fig. S9. Overlap analysis of upregulated genes in different treatment groups

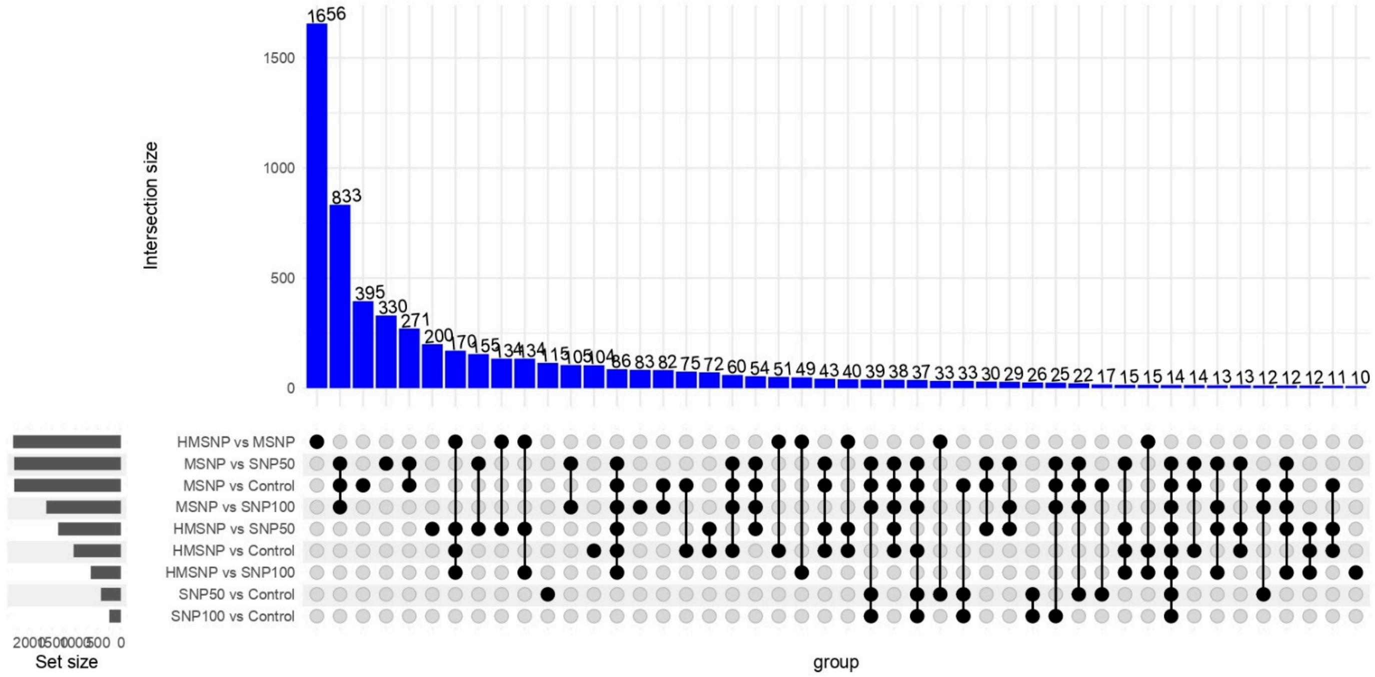

Fig. S10. Overlap analysis of downregulated genes in different treatment groups

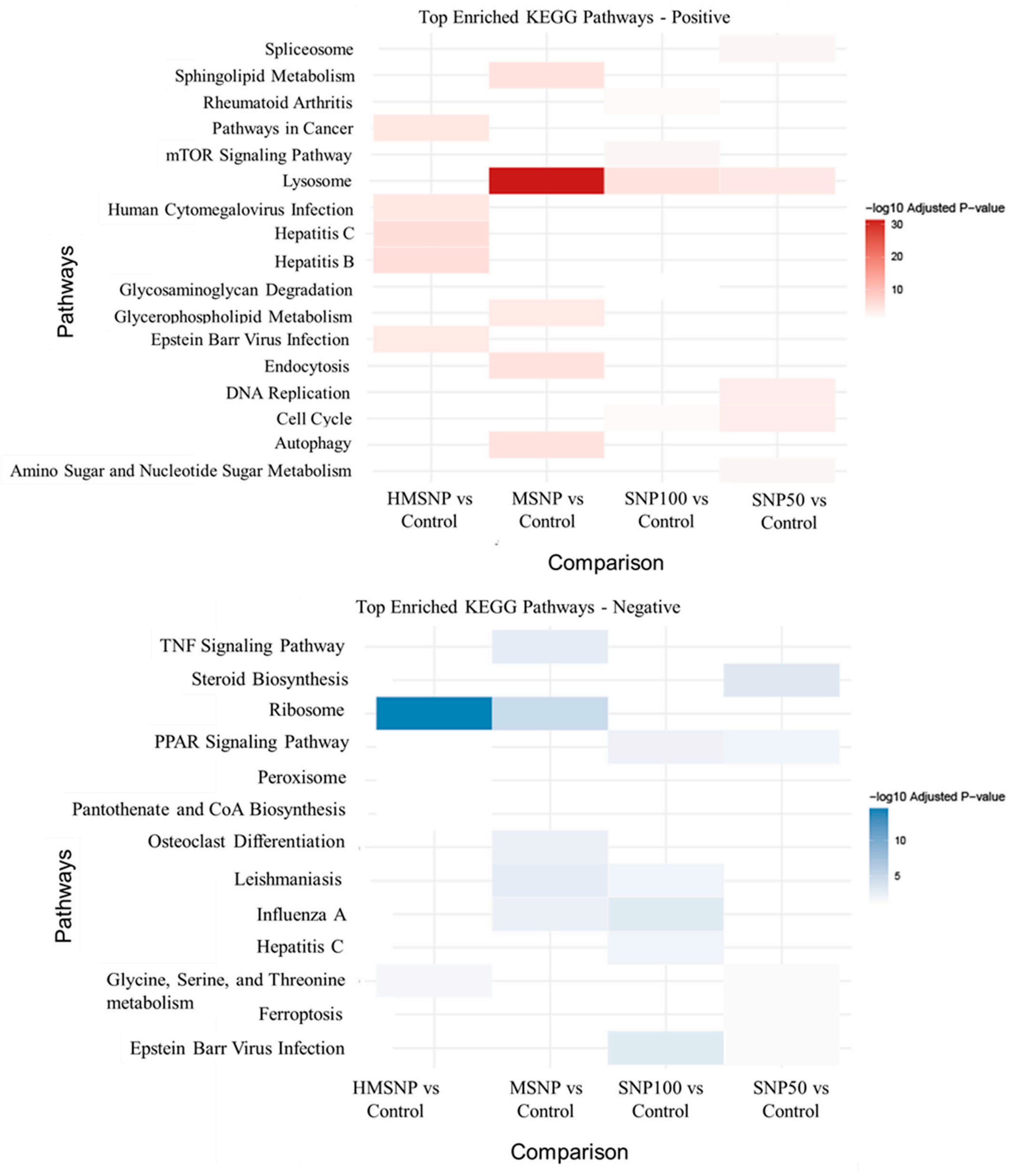

Fig. S11. KEGG pathway analyses show upregulation and downregulation of several immune pathways in the SNP treatment group compared to nontreated controls. The scales are according to the -log10 adjusted p-value.

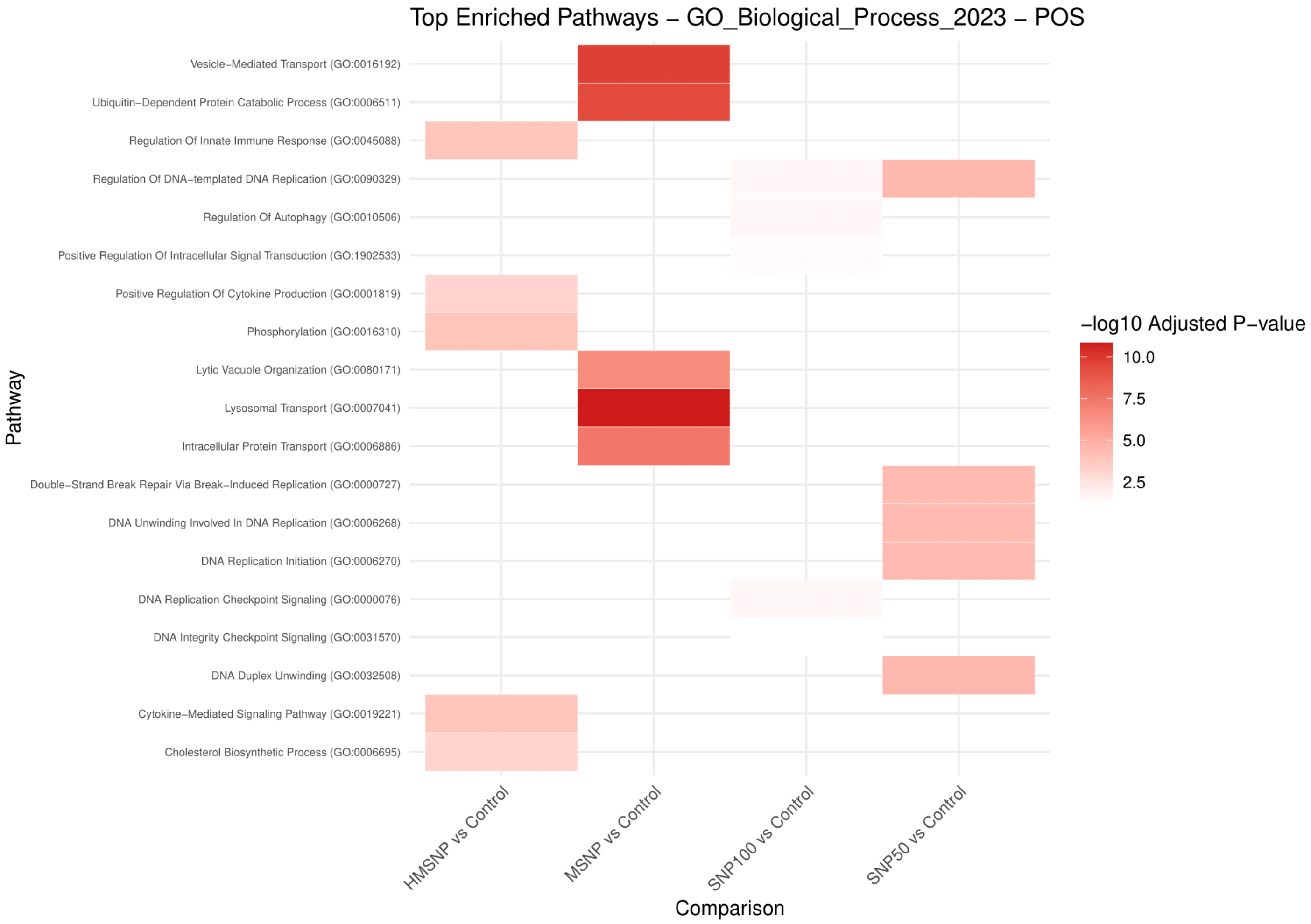

Fig. S12. Gene ontology- biological process analyses show upregulation of biological processes in the SNP treatment group compared to nontreated controls. The scales are according to the -log10 adjusted p-value.

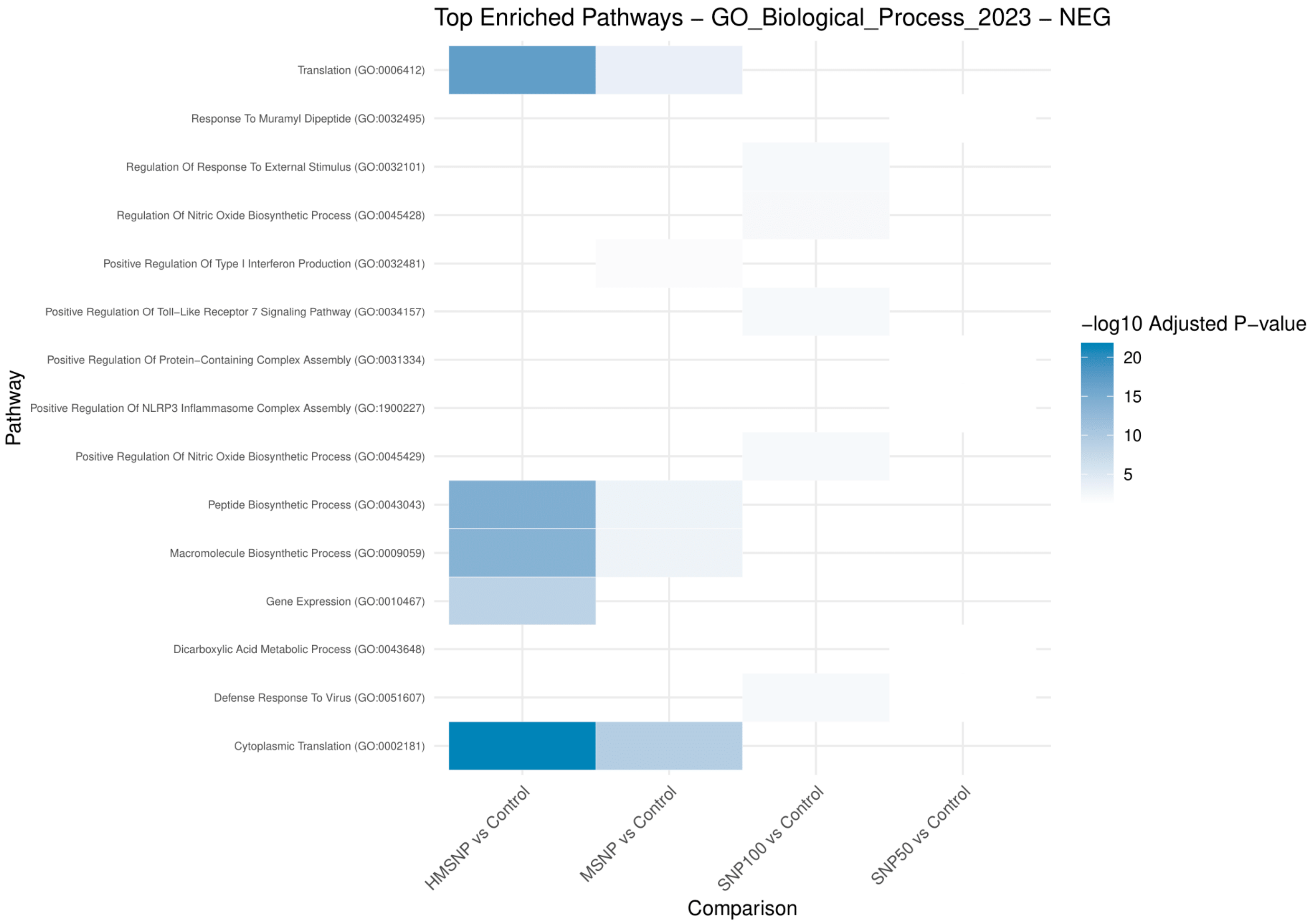

Fig. S13. Gene ontology- biological process analyses show downregulation of biological processes in the SNP treatment group compared to nontreated controls. The scales are according to the -log10 adjusted p-value.

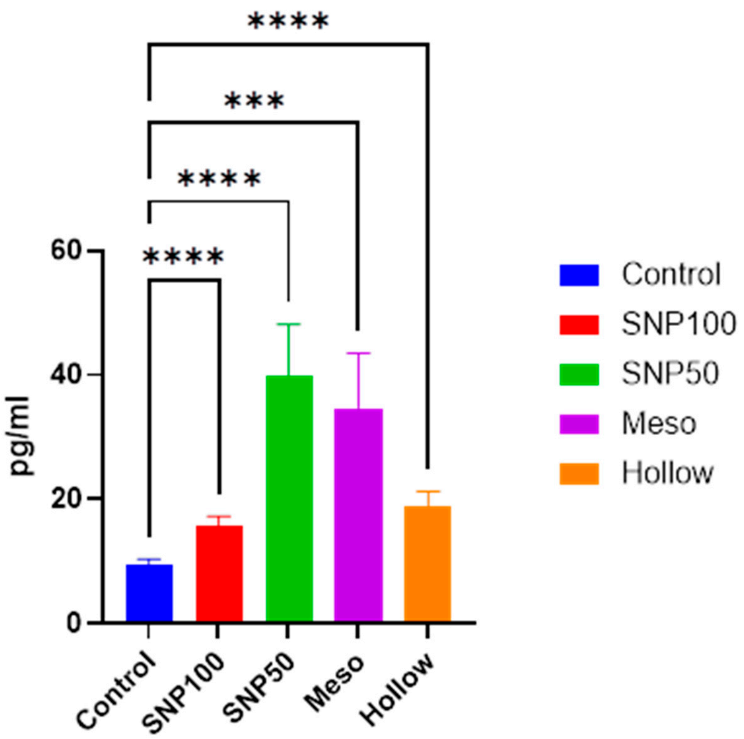

Fig. S14. TNF-α levels in the SNP-treated macrophages detected by ELISA
